# The relationship between information overload, adjustment to cancer, and level of health perception in patients with cancer: a descriptive cross-sectional study

**DOI:** 10.1590/1516-3180.2025.3521.20072026

**Published:** 2026-09-14

**Authors:** Ömer Tanriverdi, Derya Biçak Ayik

**Affiliations:** I PhD. Nursing Department, Faculty of Health Sciences, Mardin Artuklu University, Mardin, Türkiye.; II PhD. Nursing Department, Faculty of Health Sciences, Mardin Artuklu University, Mardin, Türkiye.

**Keywords:** Nursing, Patients, Information Seeking Behavior, Health Literacy, Psychosocial Adjustment, Coping Strategies, Patient Education

## Abstract

**BACKGROUND:**

Patients with cancer are frequently exposed to excessive, fragmented, and inconsistent health information; this may contribute to cognitive overload and hinder their psychological adjustment to the illness. Understanding how information overload influences coping mechanisms and perceived health status is essential for designing effective psychosocial and nursing interventions.

**OBJECTIVE:**

This study aimed to examine the relationship between information overload, psychosocial adjustment to cancer, and health perception among patients undergoing chemotherapy.

**METHODS:**

A descriptive, cross-sectional design was used. The study involved 152 patients undergoing chemotherapy at the Oncology Unit of Mardin Training and Research Hospital between June and September 2025. Data were collected using a Personal Information Form, and the Cancer Information Overload Scale, Mental Adjustment to Cancer Scale, and Health Perception Scale. Descriptive statistics, Pearson correlation analyses, and multiple linear regression were conducted, with statistical significance set at p < 0.05.

**RESULTS:**

The mean health perception score was 51.02 ± 7.44, indicating a moderate to high level of perceived health. Pearson correlation analysis revealed a positive and statistically significant relationship between information overload and health perception (r = .183; p = .024).

**CONCLUSION:**

Information overload and coping style played influential roles in shaping health perception among patients with cancer. Although excessive information can overwhelm individuals, access to accurate, comprehensible, and well-structured information appears to enhance awareness and support adaptive coping. Healthcare professionals-particularly nurses-are crucial in guiding patients through effective information management and provision of psychosocial support, ultimately improving patients’ perceived health and overall well-being.

## INTRODUCTION

Cancer is a leading cause of morbidity and mortality worldwide and continues to pose a major public health challenge. Global cancer statistics indicate a steady rise in both the incidence and mortality of the disease, with the approximately 10 million cancer-related deaths recorded in 2020 representing one in every six deaths globally.^1^ A cancer diagnosis profoundly affects both a patient’s physiological functioning and their psychological, cognitive, and social well-being.^2^ For this reason, how individuals evaluate, process, and interpret health information during the disease trajectory plays a critical role in treatment adherence and overall quality of life.

Information overload occurs when the volume or complexity of available information exceeds an individual’s cognitive capacity to process it effectively. During the complex and prolonged treatment processes characteristic of cancer care, patients are often exposed to large quantities of fragmented, inconsistent, or overly technical health information from diverse sources. This situation may lead to confusion, anxiety, indecision, and cognitive fatigue.^3^ A study has shown that information overload impairs decision-making, reduces participation in treatment, and hampers psychological adjustment to the illness.^4^ Excessive or poorly structured information may also distort patients’ health perceptions and weaken their coping abilities.

Psychosocial adjustment to cancer refers to how individuals regulate their emotional, cognitive, and behavioral responses to the diagnosis and treatment process.^5^ ^,^ ^6^ The adjustment process incorporates a wide range of reactions-from shock and fear to acceptance and active engagement. Kübler-Ross’s^7^ classic framework describes these emotional responses across the stages of denial, anger, bargaining, depression, and acceptance. High levels of adjustment are associated with active treatment participation, whereas poor adjustment is linked to anxiety, helplessness, and depression.^8^

Health perception is a multifaceted concept encompassing individuals’ beliefs, feelings, expectations, and judgments about their current health status.^9^ Understanding patients’ perception of their health is essential for planning individualized care, improving communication between patients and healthcare professionals, and supporting comprehensive care throughout the cancer trajectory, particularly in chronic and palliative care settings.^10^ A positive health perception is known to enhance coping ability, support psychological well-being, and increase treatment adherence.^11^ ^,^ ^12^ Conversely, negative health perception is associated with low motivation, heightened distress, and decreased engagement in health-promoting behaviors.

Although previous research has examined information overload, coping, and health perception separately, studies investigating their interrelationships are limited. Evidence on how information overload influences both adjustment to illness and health perception in patients with cancer is particularly scarce. A deeper understanding of these variables is essential for improving patient-centered communication, designing effective educational interventions, and strengthening psychosocial support practices in oncology care.

Therefore, in this study, we aimed to examine the relationships among information overload, psychosocial adjustment to cancer, and health perception in individuals undergoing chemotherapy, and to provide new insights into the cognitive and psychological mechanisms that shape coping in patients with cancer. Unlike previous studies that have predominantly examined information overload, coping, or health perception independently, these variables were integrated within a single conceptual framework in this study. Furthermore, we explored the potentially paradoxical role of information overload as a cognitive burden and also as a factor that may enhance awareness and perceived health. By addressing this dual role, we offer a more nuanced understanding of how patients process and adapt to health-related information during cancer treatment, thereby contributing both conceptually and clinically to the existing literature.

## METHODS

A descriptive, cross-sectional design was used in this study to examine the relationships among information overload, psychosocial adjustment to cancer, and health perception in individuals undergoing chemotherapy. The design was selected to capture the current status of these variables within a defined period and to explore their interrelationships without manipulating the study environment.

### Study design and population

The study was conducted at the Oncology Unit of Mardin Training and Research Hospital between June and September 2025. The target population consisted of adult patients diagnosed with cancer who underwent chemotherapy during the data collection period. A total of 152 patients participated in the study, all of whom completed the questionnaires through face-to-face interviews on a voluntary basis.

An a priori power analysis was conducted using G*Power 3.1 to determine the minimum sample size required for multiple linear regression analysis. Based on a moderate effect size (f² = 0.15), an alpha level of 0.05, a statistical power of 0.8, and six predictors, the minimum sample size required was calculated as 92. Because the final sample included 152 participants, the study was considered to have sufficient statistical power.

The inclusion criteria were as follows: being a voluntary participant in the study, being 18 years or older, having received at least one cycle of chemotherapy, and having sufficient cognitive capacity to respond to all questionnaire items. The exclusion criterion was having any diagnosed psychiatric disorder.

### Data collection

A structured Personal Information Form consisting of 11 items was developed by the researchers to collect participants’ sociodemographic and clinical characteristics. The form included prompts to capture demographic and clinical characteristics such as age, gender, education level, marital status, income status, cancer type, duration of illness, disease stage, information sources, year of diagnosis, and presence of psychiatric illness or medication use.

### Cancer information overload

The Cancer Information Overload Scale, originally developed by Jensen et al.,^13^ was used to measure patients’ perceived information overload regarding cancer. The Turkish version of the scale was translated and culturally adapted by İnci et al.^14^ a Cronbach’s α = 0.77 was reported by İnci̇ et al.^14^ for the Turkish adaptation. The scale contains eight items rated on a four-point Likert scale ranging from 1 (Strongly disagree) to 4 (Strongly agree), with total scores ranging from 8 to 32. Higher scores indicate greater perceived information overload. The scale demonstrated good internal consistency in the original study (Cronbach’s α = 0.87).

### Health perception

The Health Perception Scale, developed by Diamond et al.^15^ and adapted into Turkish by Kadıoğlu and Yıldız,^16^ was used to assess individuals’ perceived health status. The scale comprises 15 items across four subdimensions: control center, certainty, self-awareness, and importance of health. Items are rated on a five-point Likert scale, with negative items reverse-scored. Total scores range from 15 to 75, with higher scores indicating a more positive health perception.^16^

### Mental Adjustment to Cancer (MAC) Scale

The Cancer Coping Style Scale, which is the Turkish adaptation of the Mental Adjustment to Cancer (MAC) Scale originally developed by Watson et al.,^17^ was used to assess patients’ coping styles in response to cancer. The Turkish validity and reliability study of the scale was conducted by Natan.^18^

The scale consists of 40 items grouped into five subscales: Fighting Spirit (16 items), Anxious Preoccupation (9 items), Helplessness/Hopelessness (6 items), Fatalism (8 items), and Denial/Avoidance (1 item). Higher scores on each subscale indicate a greater tendency toward that particular coping style. Each item on the scale is rated using a four-point Likert scale: 1 (*Definitely does not apply to me*), 2 (*Does not apply to me*), 3 (*Applies to me*), and 4 (*Definitely applies to me*). Higher scores indicate a greater response in the relevant subdimension.

### Limitations of the study

The study was conducted at a single center with patients receiving treatment in the oncology department and undergoing chemotherapy. Therefore, the findings cannot be generalized to all patients with cancer. Furthermore, the study was limited by the subjective responses of patients to the data collection tools and the dimensions covered by these tools. The cross-sectional and descriptive design of the study limit the ability to establish causal relationships between variables.

### Statistical analysis

Categorical variables are summarized using number (n) and percentage (%), and continuous variables using mean ± standard deviation (Mean ± SD) and minimum-maximum values. The distribution of the total scale and subscale scores was examined using histograms and Q-Q plots; relationships between binary continuous variables that met normality were tested using two-tailed Pearson correlation (r). The significance level was set at α = 0.05.

Hierarchical multiple linear regression analysis was performed using the Health Perception Scale (total score) as the dependent variable. The Mental Adjustment to Cancer (MAC) Scale (total score) and Cancer Information Overload (total score) were entered in the first block, while age, disease duration, disease stage, and cancer type were entered in the second block. Model fit is reported using R, R², adjusted R², and analysis of variance (F). The B, SE, β, t, p, and the 95% CI values are provided for the coefficients (4). Tolerance and variance inflation factor thresholds (tolerance > 0.10; variance inflation factor < 10) were considered for multicollinearity normality of residuals (Q-Q plot) and variance homogeneity (associated with-residual scatter) were reviewed, and potential influential observations were assessed using Cook’s D and leverage. All tests were two-tailed, and p < 0.05 was considered significant.

### Ethical aspects

Prior to commencing the study, ethical approval was obtained from the Non-Interventional Ethics Committee of Mardin Artuklu University (approval number 2025/2-10, session number 2). The necessary legal permissions were obtained from Mardin Artuklu University (ethical approval) and Mardin Training and Research Hospital. Informed consent was obtained from the patients in accordance with the Declaration of Helsinki. Individuals who voluntarily agreed to participate in the study were included after their informed consent was obtained.

## RESULTS

A total of 152 patients with cancer participated in the study. The average age of the participants was 53.94 ± 15.54 years (min.: 22; max.: 85), and the average duration of the disease was 2.49 ± 1.96 years. Of the participants, 57.2% were male, 82.9% were married, 34.9% were illiterate, and 54.6% had an income that equaled their expenditure. Moreover, 25% of participants had lung cancer and 48% had stage 4 disease, indicating that a significant portion of the sample consisted of patients with advanced-stage cancer.

When information sources were examined, 47.4% of participants stated that they did not use any information sources.

In general, these findings indicate that the majority of participants were middle-aged, married individuals with low to middle income levels. A significant proportion of participants had been diagnosed with advanced-stage cancer and utilized information sources to a limited extent (Table 1).

**Table 1.** Socio-demographic and clinical characteristics of participants (N = 152)

Characteristic		Number	%
Gender	*Female*	65	42.8
	*Male*	87	57.2
Marital status	*Married*	126	82.9
	*Single*	26	17.1
Level of education	*Illiterate*	53	34.9
	*Primary school*	44	28.9
	*Secondary school*	24	15.8
	*High school*	21	13.8
	*College and above*	10	6.6
Income status	*Income is less than expenditure*	57	37.5
	*Income equals expenditure*	83	54.6
	*Income exceeds expenditure*	12	7.9
Cancer type	*Lung*	38	25.0
	*Colorectal*	13	8.6
	*Stomach*	32	21.1
	*Breast*	25	16.4
	*Pancreas*	8	5.3
	*Prostate*	17	11.2
	*Lymphoma*	14	9.2
	*Ovarian*	5	3.3
Disease stage	*Stage 1*	15	9.9
	*Stage 2*	34	22.4
	*Stage 3*	30	19.7
	*Stage 4*	73	48.0
Information sources	*Internet*	57	37.5
	*Television*	20	13.2
	*Newspaper*	3	2.0
	*None*	72	47.4
Source usage frequency	*Never*	16	10.5
	*Rarely*	25	16.4
	*Occasionally*	71	46.7
From sources	*Rarely*	32	21.1
	*Occasionally*	59	38.8
Recommendation application (*Frequently*)		43	28.3

Avg., average; Max., maximum; Min., minimum; SS, standard deviation.

The mean total score on the Participants’ Health Perception Scale was 51.02 ± 7.44, indicating that participants generally perceived their health status to be above average. When examining the subscales, the means for the Certainty, Importance of Health, Self-Awareness, and Control Center subscales were 13.26 ± 2.84, 10.20 ± 4.40, 11.88 ± 2.21, and 15.68 ± 3.65, respectively. These results indicate that individuals’ awareness of their health and their perception of control were pronounced, while the importance they attached to their health was moderate.

The average total score for the Mental Adjustment to Cancer Scale was 108.73 ± 9.66. When examining the subscales, the average score was highest for Fighting Spirit at 48.15 ± 6.17, followed by Anxious Preoccupation (24.53 ± 3.44), Fatalism (20.75 ± 4.05), Hopelessness (12.87 ± 3.54), and Denial (2.43 ± 0.90; Table 2). These findings indicate that participants adopted a more active, combative attitude in coping with cancer, with low levels of helplessness and denial.

**Table 2.** Distribution of participants’ scores on the total scale and subscales (N = 152)

Scale and subscale	Min.	Max.	Mean	SD
Health Perception Scale total score	21	75	51.02	7.44
*Certainty*	6	20	13.26	2.84
*Importance of health*	3	41	10.20	4.40
*Self-awareness*	4	15	11.88	2.21
*Control center*	8	24	15.68	3.65
Mental Adjustment to cancer total score	70	137	108.73	9.66
*Fighting spirit*	34	65	48.15	6.17
*Helplessness*	6	22	12.87	3.54
*Anxious preoccupation*	11	42	24.53	3.44
*Fatalism*	10	45	20.75	4.05
*Denial*	1	4	2.43	0.90
Cancer Information Overload Scale total score	8	32	22.41	4.83

Max., maximum; Min., minimum; SD, standard deviation.

The mean total score for the Cancer Information Overload Scale was 22.41 ± 4.83, indicating a moderate level of perceived information overload among participants (Table 2). Overall, the findings suggest that participants’ perceptions of health were positive, their coping styles were active and combative, and their levels of information overload were moderate.

Pearson correlation analysis was conducted to examine the relationships between health perception, Mental Adjustment to Cancer, and Cancer Information Overload. According to the correlation analysis results, significant relationships were found between the subscales of the Health Perception Scale and Mental Adjustment to Cancer Scale. A positive and significant relationship (r = .212; p = .009) was found between the Fighting Spirit subdimension and Self-Awareness, whereas a negative and significant relationship (r = −.192; p = .018) was found between the total score of the Health Perception Scale and Fighting Spirit. This finding indicates that as health perception increases, individuals’ use of combative approaches decrease.

The Helplessness subscale was positively and significantly correlated with the Certainty (r = .288; p < .001) and Control Center (r = .397; p < .001) subscales. A positive and strong relationship (r = .311; p < .001) was found between the total Health Perception Scale score and Helplessness subscale. This indicates that individuals experiencing uncertainty in their health perception may experience increased feelings of helplessness.

Similarly, the Anxious Preoccupation subscale showed a positive relationship with Certainty (r = .280; p < .001), Control Center (r = .281; p < .001), and the total Health Perception Scale score (r = .347; p < .001).

The Fatalism subscale showed a positive and significant relationship with Control Center (r = .326; p < .001) and the total Health Perception Scale score (r = .160; p = .049). This finding suggests that the sense of fatalism may increase as perceived control over health increases.

The Denial subscale showed a weak but significant positive correlation with the total score of the Health Perception Scale (r = .194; p = .016).

The total score for the Mental Adjustment to Cancer Scale was significantly and positively correlated with the subscale Control Center (r = .330; p < .001) and the total score for the Health Perception Scale (r = .252; p = .002).

The total score for the Cancer Information Overload Scale showed a positive and significant relationship with the subscales Certainty (r = .334; p < .001), Control Center (r = .340; p < .001), and the total score for the Health Perception Scale (r = .183; p = .024). These results indicate that individuals with high perceptions of health perception also had increased perceptions of information overload (Table 3).

**Table 3.** Examination of the relationships between the Health Perception Scale subdimensions, the Mental Adjustment to Cancer Scale, and the Cancer Information Overload Scale

Health Perception Scale							
Scale and or subscale		Certainty	Importance of Health	Self-Awareness	Control Center	Health Perception Scale	Cancer Information Overload
						Total Score	Total score
Fighting spirit	r	−.109	.125	.212	−.105	.044	−.192
	p	0.182	0.125	**0.009**	0.198	0.592	0.018
Helplessness	r	.288	−.197	−.167	.397	.139	.311
	p	< 0.001	0.015	**0.040**	< 0.001	0.088	< 0.001
Anxious preoccupation	r	.280	.077	.191	.281	.347	.166
	p	< 0.001	.346	.018	< 0.001	< 0.001	.041
Fatalism	r	.104	−.125	−.073	.326	.104	.160
	p	0.204	0.126	0.371	< 0.001	0.202	0.049
Denial	r	.140	−0.054	−.089	.152	.070	.194
	p	0.086	0.509	0.278	0.061	0.394	0.016
(MAC) Scale total score	r	.192	−.022	.103	.330	.252	.136
	p	**0.018**	0.786	0.207	< 0.001	**0.002**	0.095
Cancer Information Overload total score	r	.334	−.119	−.136	.340	.183	1
	p	< 0.001	0.145	0.095	< 0.001	0.024	-

Overall, significant relationships were found between certain subdimensions of health perception and both coping styles with cancer and information overload. This highlights that individuals’ health perception is an important psychological variable that influences their coping styles for cancer and their level of exposure to information.

Multiple linear regression analysis showed that the Mental Adjustment to Cancer (MAC) Scale (total score) and the Cancer Information Overload (CIO) Scale (total score) were significant predictors of health perception (Table 4). Mental Adjustment to Cancer (β = 0.236; t = 3.004; p = 0.003). A significant positive association was observed between Mental Adjustment to Cancer and health perception. This finding indicates that as individuals develop more active, aware, and adaptive coping styles toward cancer, their health perception also increases. In other words, having more positive coping strategies contributes to an individual feeling healthier.

**Table 4.** Multiple linear regression coefficients for Health Perception

Associated predictor	Beta	Standard error	Standardized beta	t	p	95% CI	
Fixed	29.122	6.943	-	4.194	**< 0.001**	15.401	42.843
(MAC) Scale (total score)	0.182	0.061	0.236	3.004	**0.003**	0.062	0.302
(CIO) Scale (total score)	0.281	0.124	0.183	2.273	**0.024**	0.037	0.526
Age	−0.069	0.038	−0.144	−1.810	0.072	−0.144	0.006
Duration of illness	−0.191	0.296	−0.050	−0.643	0.521	−0.776	0.395
Disease stage	−1.919	0.549	−0.271	−3.494	**0.001**	−2.995	−0.843
Cancer type	0.431	0.275	0.123	1.566	0.119	−0.108	0.970

Information Overload (β = 0.183; t = 2.273; p = 0.024). The information overload variable and health perception also showed a significant positive association (Table 4). This finding suggests that exposure to information does not always have a negative effect; on the contrary, access to sufficient information can increase an individual’s awareness and strengthen their health perception. Therefore, the quality of information and how it is interpreted may be more decisive than the quantity of information.

Disease Stage (β = −0.271; t = −3.494; p = 0.001). Disease stage was found to be a strong variable with a significant negative association with health perception. As the disease stage progressed, individuals’ health perception significantly decreased (Table 4). This finding shows that patients with advanced-stage cancer experience a decline in their health perception due to physical fatigue, treatment side effects, and the chronic nature of the disease.

### DISCUSSION

In this study, we examined the relationships between information overload, psychosocial adjustment to cancer, and health perception in patients undergoing chemotherapy. The findings provide important insights into how cognitive and emotional processes interact during the cancer trajectory, and highlight the need for patient-centered communication strategies in oncology care.

The sociodemographic characteristics of the sample showed that most participants were middle-aged or older, married, with low educational and income levels. These characteristics are consistent with previous studies conducted in Türkiye and similar regions, where educational limitations and socioeconomic constraints often restrict patients’ access to reliable cancer-related information.^19^,^20^ Notably, nearly half the participants reported not using any information sources, which may reflect limited health literacy and technological barriers. Such limitations can exacerbate uncertainty and contribute to confusion during the treatment process.

Significant associations were found between health perception and coping styles. The positive relationship between self-awareness and fighting spirit suggests that individuals who have a clearer understanding of their illness tend to engage in more active coping behaviors. This is consistent with previous research in which higher illness awareness was demonstrated to promote adaptive coping and emotional regulation among patients with cancer.^21^,^22^ Interestingly, however, the negative correlation observed between overall health perception and fighting spirit may indicate that individuals who perceive their health more positively may rely less on combative coping strategies and may instead adopt more acceptance-oriented approaches. This could reflect a shift in coping priorities among individuals with chronic or advanced-stage illness.

The findings also revealed significant relationships between maladaptive coping strategies such as helplessness, anxious preoccupation, and fatalism, and the subdimensions of health perception. The strong associations found between helplessness and both certainty and control center suggest that a perception of less control over health may heighten feelings of helplessness; this finding is consistent with those of previous studies linking negative perceptions of illness to psychological distress.^23^,^24^ Similarly, the positive relationship demonstrated between fatalism and both control center and total health perception may indicate that some patients interpret their illness within a broader spiritual or destiny-oriented framework. A study conducted among the American public demonstrated that fatalistic beliefs were associated with reduced cancer information-seeking behavior.^25^

A notable finding of this study was the positive association observed between information overload and health perception (r = .183; p = .024). This finding is partially consistent with those of previous studies that suggest that increased access to health information can enhance patients’ awareness and engagement in their care.^19^,^26^ However, this finding contrasts with the evidence that is predominant in the literature, namely, that information overload is often associated with negative outcomes such as confusion, increased anxiety, and difficulties in decision-making.^3^,^4^ This discrepancy may be explained by differences in health literacy levels, the quality and structure of information, and the clinical context in which patients process information.^19^,^26^ Although information overload is often conceptualized as a negative cognitive state associated with confusion, anxiety, and reduced decision-making capacity,^3^,^4^ our results suggest a more nuanced dynamic. Participants with higher health perception also reported greater perceived information load. One possible explanation is that patients who seek more information may become more aware of their health status, leading to increased sensitivity to the amount of information they encounter. Another interpretation is that access to accurate and comprehensible information may enhance individuals’ understanding of their illness, even when the amount of information feels overwhelming. This supports the findings in the literature in which health literacy is demonstrated to mitigate the negative effects of information overload by improving comprehension and fostering a sense of empowerment.^26^

The results of the regression analysis further revealed that coping style and information overload were significant positive predictors of health perception, but that disease stage was a negative predictor. As expected, individuals with more advanced disease reported lower perceptions of their health. This aligns with the established evidence that advanced-stage cancer is associated with heightened symptom burden, functional decline, and reduced quality of life.^2^ The positive predictive effect of coping style reinforces the role of psychosocial adaptation in shaping health-related perceptions and underscores the importance of supportive interventions that promote adaptive coping strategies.

Overall, the interplay between information overload, coping, and health perception identified in this study emphasizes the critical role of healthcare professionals, particularly nurses, in structuring and tailoring patient education. Providing clear, comprehensible, and individualized information may help reduce confusion while strengthening patients’ psychological resources for coping with the illness. Improving access to reliable information and supporting patients’ cognitive and emotional processing of this information can mitigate the negative consequences of overload, enhance adjustment, and ultimately contribute to improved health perception and well-being. From a clinical perspective, these findings highlight the importance of reducing information overload as well as optimizing the quality, clarity, and personalization of information provided to patients. Oncology nurses and other healthcare professionals play a crucial role in filtering, structuring, and delivering information in a manner that aligns with patients’ cognitive capacity and emotional needs.

Moreover, routine assessment of patients’ information needs and coping styles may help identify individuals at risk of cognitive overload or maladaptive adjustment. Accordingly, tailored educational and psychosocial interventions can enhance patients’ understanding, support adaptive coping strategies, and ultimately improve health perception and overall well-being.

## CONCLUSION

We demonstrated significant relationships among information overload, psychosocial coping styles, and health perception in patients undergoing chemotherapy in this study. The findings highlight that both the amount and complexity of cancer-related information, as well as the ways in which individuals cope with their illness, play influential roles in shaping their overall health perception. Higher levels of information overload were associated with increased health perception, but this relationship appears to reflect a greater level of awareness of illness rather than a negative cognitive burden. Furthermore, adaptive coping styles were found to enhance perceived health, whereas advanced disease stage was associated with lower health perception.

These results indicate the importance of providing structured, comprehensible, and patient-centered information to individuals with cancer. Healthcare professionals, particularly nurses, have a critical role in guiding patients through the overwhelming quantity of medical information and supporting their psychological adjustment throughout treatment. Strengthening patients’ coping capacities and ensuring that they receive accurate and relevant information may help improve their understanding of the illness, reduce uncertainty, and promote more positive health perceptions.

### Implications for practice

Oncology nurses should tailor educational materials to patients’ cognitive and emotional needs to prevent unnecessary information burden. Interventions that promote adaptive coping strategies may enhance patients’ psychological resilience and health perception. Systematic assessment of patients’ information needs, health literacy, and coping patterns should be integrated into routine oncology care.

## Data Availability

The datasets generated and/or analyzed during the current study are not publicly available because they contain information that could compromise the privacy of the participants but are available from the corresponding author, Ömer Tanriverdi, on reasonable request.
